# Experiences of participating in a problem-solving intervention with workplace involvement in Swedish primary health care: a qualitative study from rehabilitation coordinator's, employee's, and manager's perspectives

**DOI:** 10.1186/s12889-023-15899-y

**Published:** 2023-05-24

**Authors:** Ida Karlsson, Lydia Kwak, Iben Axén, Gunnar Bergström, Ute Bültmann, Kristina Holmgren, Elisabeth Björk Brämberg

**Affiliations:** 1 Institute of Environmental Medicine, Unit of Intervention and Implementation Research for Worker Health, Karolinska Institutet, Stockholm, Sweden; 2 Department of Occupational Health Sciences and Psychology, Faculty of Health and Occupational Studies, University of Gävle, Gävle, Sweden; 3 Department of Health Sciences, Community and Occupational Medicine, University of Groningen, University Medical Center Groningen, Groningen, The Netherlands; 4 Department of Health and Rehabilitation, Institute of Neuroscience and Physiology, Sahlgrenska Academy, University of Gothenburg, Gothenburg, Sweden

**Keywords:** Common mental disorders, Depression, Anxiety, Adjustment disorder, Problem-solving, Sickness absence, Primary care

## Abstract

**Background:**

Work-directed interventions that include problem-solving can reduce the number of sickness absence days. The effect of combining a problem-solving intervention with involvement of the employer is currently being tested in primary care in Sweden for employees on sickness absence due to common mental disorders (PROSA trial). The current study is part of the PROSA trial and has a two-fold aim: 1) to explore the experiences of participating in a problem-solving intervention with workplace involvement aimed at reducing sickness absence in employees with common mental disorders, delivered in Swedish primary health care, and 2) to identify facilitators of and barriers to participate in the intervention. Both aims targeted rehabilitation coordinators, employees on sickness absence, and first-line managers.

**Methods:**

Data were collected from semi-structured interviews with participants from the PROSA intervention group; rehabilitation coordinators (*n* = 8), employees (*n* = 13), and first-line managers (*n* = 8). Content analysis was used to analyse the data and the Consolidated Framework for Implementation Research was used to group the data according to four contextual domains. One theme describing the participation experiences was established for each domain. Facilitators and barriers for each domain and stakeholder group were identified.

**Results:**

The stakeholders experienced the intervention as supportive in identifying problems and solutions and enabling a dialogue between them. However, the intervention was considered demanding and good relationships between the stakeholders were needed. Facilitating factors were the manual and work sheets which the coordinators were provided with, and the manager being involved early in the return-to-work process. Barriers were the number of on-site meetings, disagreements and conflicts between employees and first-line managers, and symptom severity.

**Conclusions:**

Seeing the workplace as an integral part of the intervention by always conducting a three-part meeting enabled a dialogue that can be used to identify and address disagreements, to explain CMD symptoms, and how these can be handled at the workplace. We suggest allocating time towards developing good relationships, provide RCs with training in handling disagreements, and additional knowledge about factors in the employee’s psychosocial work environment that can impair or promote health to increase the RCs ability to support the employee and manager.

**Supplementary Information:**

The online version contains supplementary material available at 10.1186/s12889-023-15899-y.

## Introduction

Common mental disorders (CMDs), i.e. depression, anxiety and adjustment disorder, affect approximately one in six individuals in the European Union [1]. These disorders can influence the ability to work, and cope with everyday stressors [1, 2]. Persons with CMDs experience more sickness absence (SA), their SA episodes are also longer compared with other diagnoses and have an increased risk of recurring SA [3].

Clinical interventions for CMDs, such as cognitive behavioural therapy usually result in symptom reduction, but symptom reduction is not always followed by a reduced number of SA days [4, 5]. A recent Cochrane review showed that combining a clinical intervention, such as cognitive behavioural therapy or problem-solving with a work-directed intervention (e.g. which involves the workplace, work conditions, occupational case management strategies and/or stakeholders) probably reduces the amount of SA during the first year by up to 25 days compared to care-as-usual [6]. A problem-solving intervention combined with a work directed intervention has also been shown to reduce the time to partial return to work (RTW) [7] and time to first RTW [8, 9]. The evidence regarding full RTW is inconclusive [7, 9]. Even if the results of combining a problem-solving intervention with a work directed intervention are promising [10, 11] these studies have been conducted in countries (mainly the Netherlands) with a different social insurance system than Sweden, and in the occupational health services. Currently, we lack knowledge about the effectiveness of combining a problem-solving intervention with a work-directed intervention when conducted in the Swedish primary health care system (PHC).

It is recommended that complex interventions should be evaluated by means of an effectiveness and process evaluation which includes stakeholder perspectives [12]. Building on these recommendations, the current study investigates the experiences of participating in a problem-solving intervention with workplace involvement (PSI-WPI) aimed at reducing SA due to CMDs in the Swedish PHC – PROSA. In PROSA, the PSI-WPI includes a stepwise dialogue between a health care professional, an employee on SA due to CMDs, and the employee’s first-line manager [13]. In recent years, the Swedish PHC has started to provide work-directed interventions to reduce SA among employees with CMDs by involving the first-line manager in the RTW process. Despite this, there are no policies or guidelines for the PHC describing the content of such interventions [14]. The aim of PROSA (besides evaluating the effectiveness of the PSI-WPI on SA) is therefore to test a process that can help the PHC to structure the support given to employees on SA due to CMDs. This study specifically addresses contextual factors, in this case the experiences of participating in the PSI-WPI, as well as facilitators and barriers. To the best of our knowledge, no previous such studies has been carried out in a PHC setting. Understanding these factors is important to be able to interpret why an intervention is or is not effective, and to guide further adaptation of the intervention before a large-scale roll-out [15–17].

### The problem-solving intervention with workplace involvement

The effect of a PSI-WPI in a Swedish PHC setting is currently being evaluated in a cluster randomized controlled trial aimed at reducing SA due to CMDs (PROSA) [13]. The problem-solving principles have been described by Nezu and Nezu (2018). They integrate the patient’s own ideas while being supported by a health care professional [18]. The PSI-WPI involves the employee and their first-line manager and is based on a participatory approach [18, 19].

**Fig. 1:**
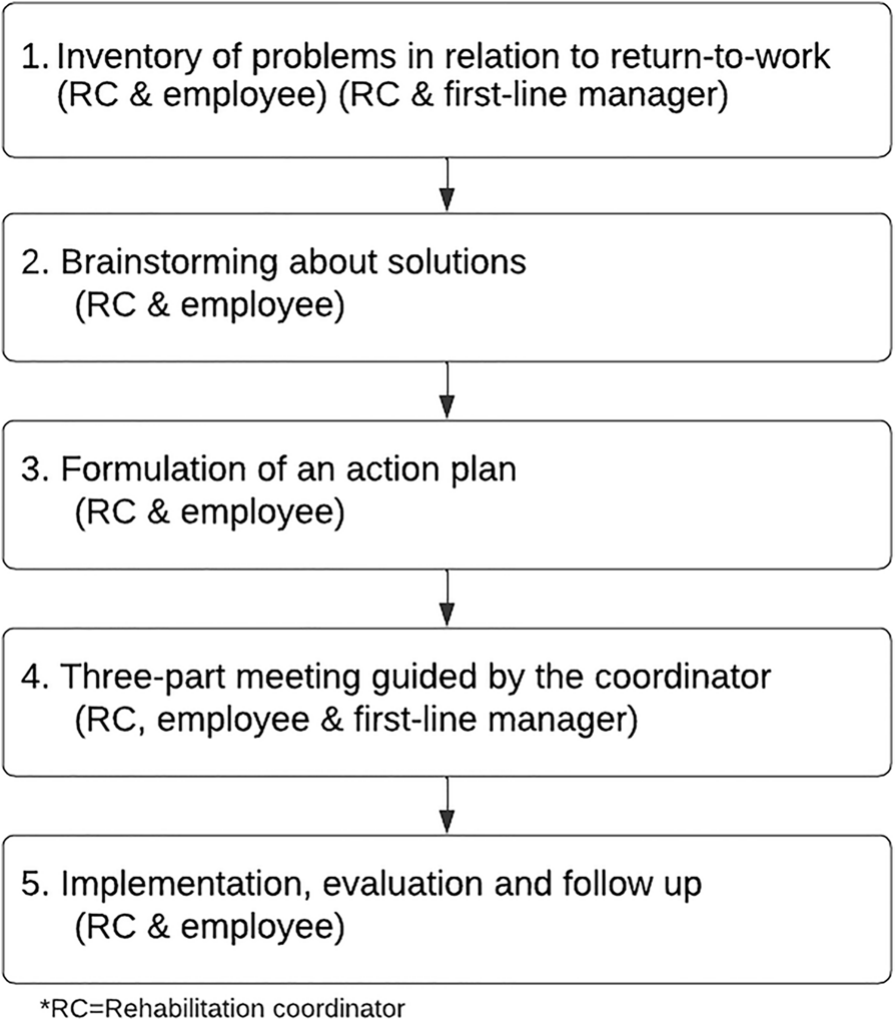
The 5-step problem-solving intervention with workplace involvement. Figure adapted from Björk Brämberg et al. [13]

In PROSA, the PSI-WPI was delivered by a rehabilitation coordinator (RC) in at least two and up to five sessions. The RCs received instructions to plan the sessions together with the employee while adhering to the employee’s health status and current symptoms. For example, steps one and two, or one to three could be delivered during the same session. The RCs received a two-day training in delivering the PSI-WPI, led by a licensed psychologist. The RCs were provided with a manual and worksheets. The first step involves an inventory of problems and/or opportunities related to RTW conducted by the RC and the employee (approx. 45 min). Thereafter, the RC contacts the first line manager to conduct a short problem-inventory to assess whether the first-line manager was aware of the employees’ health problems (approx. 15 min). The second step includes a brainstorming session between the RC and the employee. During this session, possible solutions are identified, and topics prepared that will be discussed during the meeting with all three stakeholders. The third step involves the RC and the employee. The aim of this step is to form an action-plan based on the identified problems and solutions and to identify the support needed to implement them. Step four concerns an on-site ‘three-part meeting’ involving the RC, the employee, and the first-line manager (approx. 45–60 min). The RC facilitates the meeting and fosters a dialogue between the employee and the first-line manager. Step five consists of implementing the action plan and evaluating the results, conducted by the RC and the employee (Fig. 1). For further details, see the PROSA study protocol [13].

This study will add a stakeholder perspective to the current knowledge base by exploring the experiences of the RCs, employees, and first-line managers, and looking at factors which facilitates or hinders participation in the PSI-WPI. Their experiences will provide information that can be used to support the use and further adaptions of the PSI-WPI in the PHC context.

### Aim

This study had a twofold aim: 1) to explore the experiences of participating in a problem-solving intervention with workplace involvement aimed at reducing sickness absence in employees with common mental disorders, delivered in Swedish primary health care, and 2) to identify facilitators of and barriers to participate in the intervention. Both aims targeted rehabilitation coordinators, employees on sickness absence, and first-line managers.

## Methods

### Study design

A qualitative study design was used to explore the stakeholders’ perspectives. Data were collected by means of semi-structured interviews with participants from the intervention group in PROSA. They were analysed by content analysis [20] guided by the Consolidated Framework for Implementation Research (CFIR) [15]. The reporting of the study follows the recommendations by Tong et al. [21].

### The consolidated framework for implementation research

The CFIR builds on several implementation frameworks and provides a structure for systematically assessing experiences, facilitators, and barriers. CFIR contains five domains: intervention characteristics, outer setting, inner setting, the characteristics of the individual and the process of implementation [15]. In the present study, CFIR was used to guide the development of the interview guides, and the data analysis.

Four of the CFIR domains were applied in the following manner: 1) Intervention characteristics, refers to attributes related to the PSI-WPI as perceived by the RCs, employees, and first-line managers. This includes the RCs perceptions of the structure and quality of the intervention and employees’ and first-line managers’ experiences of receiving the intervention. 2) Outer setting refers to attributes related to the outer context. This includes the employee’s needs, and facilitators of and barriers to meeting those needs from the perspectives of the RC, employee, and first-line manager. This also includes the three stakeholders’ reflections on participating in the three-part meeting. 3) Inner setting refers to the PHC setting as experienced by the RCs. This includes whether the norms and values of the PHC align with the PSI-WPI, the RCs’ satisfaction with coordinator services before working with the PSI-WPI, and willingness to adapt to a new work method. 4) Characteristics of the individual refers to knowledge and beliefs about the intervention, which is described from the perspectives of the RC, the employee, and the first-line manager. The fifth domain “implementation process” was not included because the aspects related to this domain i.e., planning and executing are not the focus of the current study.

### Setting

In Sweden, PHC is the first line of psychiatric care for persons with CMDs [22]. Physicians are responsible for assessing work ability and issuing medical certificates. The Swedish Social Insurance Agency decides if the employee is entitled to SA benefits. Benefits are mainly funded by taxes, but employers are responsible for the first two weeks of SA (except for a first qualification day). If longer SA is needed, it is paid by The Swedish Social Insurance Agency. Sickness absence for ≤ seven consecutive days does not require a medical certificate, but the employee is obliged to send a medical certificate to the employer after day seven. If the employee is expected to be on SA for more than 60 days, the employer is obliged to draw up a RTW plan [23]. In 2019, the Swedish government introduced legally required coordinator services for employees on SA [24]. Care as usual for employees on SA due to CMDs usually consists of cognitive behavioural therapy and/or pharmacological treatment. However, the waiting time for treatment can be long due to a lack of specialist psychiatric health care personnel [22, 25].

### Procedure

Three interview guides were developed for RCs, employees and first-line managers respectively. The interview guides were developed based on a study by Holmlund et al. [26] which investigated barriers and facilitators in relation to the coordination of RTW among employees on SA due to CMD in a PHC setting. These interview guides were based on the CFIR framework and were specific for each stakeholder. For the purpose of the current study, the interview questions in each guide were revised to specifically address experiences of participating in PSI-WPI. Each guide started with a description of the PSI-WPI and questions about participant characteristics followed by open-ended questions based on the CFIR [see Additional file 1]. The interviews with employees and first-line managers also contained questions assessing ethical aspects of the PSI-WPI. The analysis and findings from these questions will be reported elsewhere. The interviews were conducted by the first author IK who is a registered nurse and doctoral student, and two assistants. One of these has a doctoral degree in social work, and the other a master’s degree in occupational therapy. All interviewers had previous experience of qualitative interviewing. The principal investigator (EBB) had start-up meetings with the interviewers before the interviews were conducted, to ensure that a common interview technique was used, for example regarding probing questions or the handling of sensitive matters. Follow-up sessions were held on demand. The interviewers did not have any previous contact with the participants.

The interviews were conducted between December 2019 and October 2020. RCs were interviewed in person at their workplaces December 2019 – January 2020. Employees were interviewed April – September 2020, 12-months after their inclusion in the study. First-line managers were interviewed May – October 2020, 12-months after their employees’ inclusion in the study. The interviews with employees and first-line managers were conducted by telephone due to restrictions related to the Covid-19 pandemic. All interviews were recorded digitally and transcribed verbatim. The interviews with RCs lasted approx. 50 min (39–60 min). The first part of the interviews with employees (used for this study) lasted approx. 25 min (15–40 min) and with first-line managers approx. 23 min (8–30 min). The length of interviews varied because the number of questions for different stakeholders differed. Each audio file was cross-checked for accuracy and identifying information was removed to ensure confidentiality before the data were analyzed. It was decided that approximately 30 interviews would be sufficient, approximately 10 from each group [27].

**Fig. 2:**
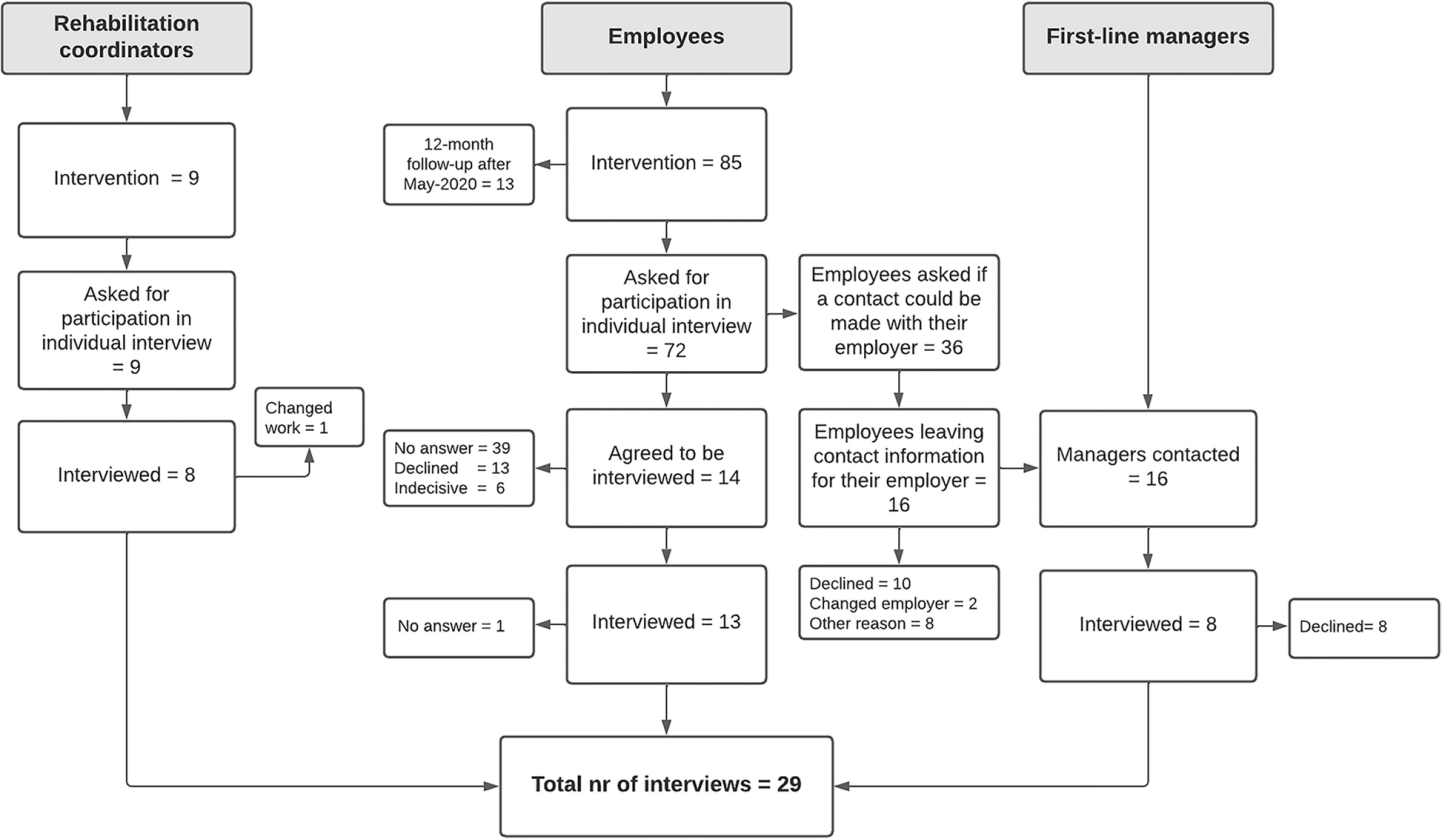
Flow-chart of recruitment and inclusion of rehabilitation coordinators, employees and first-line managers

On joining the study, all RCs, and employees, regardless of randomisation, received information about a forthcoming interview. A few weeks before the interviews took place, the RCs and employees from the intervention group received specific information related to the interview and were invited to participate. Each employee was called a maximum of three times. If they responded, they were asked to participate in an individual interview. During the call, the interviewer asked the employee for permission to contact their first-line manager. This recruitment process resulted in 29 individual interviews (Fig. 2).

### Data analysis

The data were analysed using qualitative content analysis [20] in the following steps: 1) IK listened to all audio files and read the transcripts several times to get an overall understanding of the content. EBB read all transcripts and LK read nine transcripts; 2) transcripts were explored through inductive open coding by reading the transcripts line by line and highlighting meaning units related to the aim in each interview. The data analysis software program Nvivo; version 12 was used to sort the data. Meaning units were then transferred from Nvivo to Excel for condensation; 3) meaning units were condensed descriptively by a description close to the text; 4) condensed meaning units were interpreted searching for the latent meaning and given a code; 5) codes were compared and those with similar content were combined into sub-themes (*N* = 85). After the fifth step, the analysis took a deductive approach by grouping each sub-theme according to the CFIR domains. Each CFIR domain was given a description of what it included and which stakeholder context the domain described. Using these domain descriptions, all sub-themes were categorized. A sub-theme could only fit into one domain. A number of sub-themes were found to overlap domains and the authors IK, LK and EBB reviewed those sub-themes and decided on the appropriate domain based on the most suitable contextual placement according to the domain description; 6) one theme describing the experiences of participating in the PSI-WPI was created for each CFIR domain based on the latent meaning of the codes in that sub-themes; 7) in each of the four domains, facilitators of and barriers to participating in the PSI-WPI were identified. The analysis was conducted by IK under the supervision of EBB and LK. During the later phase of the analysis, the themes, and the grouping of the sub-themes according to the CFIR domains were discussed and reviewed with all authors. Quotations were used to illustrate the link between the data and the description of the findings, identification of each stakeholder and by a participant code.

### Ethical considerations

The study was approved by the Swedish Ethical Review Authority 2017–06-21, reference number 496–17. Informed consent was obtained from all participants. The study follows the recommendations for research on human subjects as declared in the Helsinki declaration [28].

## Results

An overview of participant characteristics is given in Table 1. At baseline, the main reason for SA was a diagnosis of anxiety, stress, or depression. When comparing the baseline characteristics of the interviewed employees (*n* = 13) with PROSA study participants i.e. those who responded to the baseline questionnaire (*n* = 175) no significant differences were found regarding age, education, exhaustion assessed by the self-reported exhaustion scale [29] depression, or anxiety assessed by the Hospital Anxiety and Depression scale [30]. The stakeholders’ experiences of participating in the PSI-WPI is described in the results section. An overview of the results including CFIR domains, themes describing experiences, facilitators and barriers are presented in Table 2.

**Table 1:** Participant characteristics

	***Rehabilitation coordinators*** *n* = *8*	***Employees*** *n* = *13*	***First-line managers*** *n* = *8*
***Age in years,**** mean (range)*	57 (39–68)	44 (22–60)	46 (34–61)
***Gender***			
*Female*	8	13	4
*Male*			4
***Education***			
*Up. Sec. School*		8	1
*University*	8	5	7
***Rehabilitation coordinator basic profession***			
*Nurse*	5		
*Occupational therapist*	2		
*Physiotherapist*	1		
***Work sector***			
^a^*Public sector*	8	6	4
*Private sector*		5	4
^b^*State*		1	
*Other*		1	
***Sick leave at 12 months follow-up***			
*Returned to work*		10	
*On sick leave*		3	
***First-line manager responsibility***			
< *10 employees*			2
*10–20 employees*			4
> *20 employees*			2

^a^Public refers to work sector within regions and municipalities

^b^State refers to authorities

**Table 2:** An overview of CFIR domains, themes, stakeholders, and facilitators of and barriers to the PSI-WPI

CFIR domain	Theme (experiences)	Stakeholder	Facilitators	Barriers
**Intervention characteristics**	*The PSI-WPI supports the RC and employee by providing a structure for RTW despite the time and demands put on them*	RC	-The manual and worksheets give a clear work description of how to provide the PSI-WPI	-The extensiveness of the manual
		Employee	-The structured process of PSI-WPI helps the employees to actively participate -PSI-WPI offers the possibility to learn how to identify problems and solutions	-The number of on-site meetings to attend
		First-line manager	-PSI-WPI provide support from the RC with medical knowledge and what can be done for the employee from a rehabilitation point of view -The structure of PSI-WPI facilitates early involvement in the employees RTW process	-Needing to attend meetings on-site at the PHC
**Outer setting**	*The PSI-WPI supports employees’ needs by establishing a dialogue*	RC	-Participation in PSI-WPI was facilitated by good relationships between the stakeholders	-Disagreements or conflicts between the employee and first-line manager
		Employee	-Having confidence in the first-line manager -Receiving the first-line managers acknowledgement towards the reason for SA -Participation in PSI-WPI was facilitated by good relationships between the stakeholders -Having influence over the time and venue of the three-part meeting	-Not having the first-line manager attending the three-part meeting -Disagreements or conflicts with the RC and/or first-line manager
		First-line manager	-Participation in PSI-WPI was facilitated by good relationships between the stakeholders -Having influence over the time and venue of the three-part meeting	-Disagreements or conflicts with the employee
**Inner setting**	*The structured work method and a shared vision of how to work with SA at the PHC helps to establish the role of the RC*	RC	-The lack of a structured work method for supporting RTW caused a need for change -Having a shared vision at the PHC about how to reduce SA by seeing the workplace as an integral part of the rehabilitation -Being able to set-a-aside time for employees in the PSI-WPI -Receiving support from the principal investigator when needed	-The time-consuming aspect of the intervention i.e. PSI-WPI often took more time than care-as-usual, involves more meetings with the employee and the first-line manager and always involves the first-line manager -Juggling split roles i.e. working as an RC and as a health care professional and sometimes seeing the employee in both roles -Not having sufficient support from the PHC
**Characteristics of the individual**	*The PSI-WPI created a bridge between the PHC and the workplace, conditioned by good relationships*	RC	-The PSI-WPI made the RCs feel appreciation of the RC role	-Symptom severity of the employee influenced RCs belief in their ability to support the employee in the PSI-WPI
		Employee	-The supportive relationship with the RC increased the motivation to participate -During the meetings with the RC they were asked supportive questions which enabled reflection	-Experiences of severe symptoms was a barrier to participate in the PSI-WPI
		First-line manager	-During the three-part meeting the RC asked supportive questions -It was helpful to become involved early in the employee’s RTW process	-Feeling that the PSI-WPI took more time than the workplace service-as-usual -The characteristics of the workplace could influence the possibility to offer work accommodations

*Abbreviations*: *CFIR* Consolidated Framework for Implementation Research, *PHC* Primary Health Care, *PSI-WPI* Problem-solving intervention with workplace involvement, *RC* Rehabilitation coordinator, *RTW* Return to work, *SA* Sickness absence

### Intervention characteristics

#### The PSI-WPI supports the RC and employee by providing a structure for RTW despite the time and demands put on them

RCs felt that the PSI-WPI provided them with support in how to assess employees’ needs during the RTW process. They emphasized that the manual and worksheets were important tools when using the problem-solving approach. The RCs adapted the material provided to employees’ needs to create a feeling of having a conversation instead of an interview. “*I have understood this material (the manual) as if I kind of could follow… Because it’s a conversation, I don’t feel altogether comfortable interviewing when I talk to a patient, rather I feel confident if we can have a conversation where we’re talking on the basis of these different points*” (RC 7). On the other hand, some RCs felt that the manual was long and took more time than care-as-usual and some asked for a short version of the manual including only the primary content. Some RCs struggled with conflicts between the employee and first-line manager and would have liked the PSI-WPI to provide more support in dealing with conflicts and non-functioning relationships.

The employees described taking part in the PSI-WPI chiefly as something positive that made them take the lead, or at least become involved in identifying problems and finding solutions to their problems. By taking an active role in their own RTW process, employees felt supported in formulating their thoughts and their feelings. This helped them deal with their problems and decide how to move forward “*It was important for me to get help to, to formulate my thoughts, maybe I could have solved it on my own, but eh I kind of told myself … it was when I told her that I understood the meaning … ehm … and then she also helped guide me forward, how I would … would deal with myself and certain things I otherwise would prefer to sweep under the carpet, okay, I really have to deal with this to be able to move on, to feel better”* (Employee 13). The support of the RC increased the employees’ motivation to prioritise and implement certain solutions. The support of the RC also motivated employees to discuss concerns around SA and RTW. However, some employees reported that it was sometimes difficult to attend all the required meetings, which made them feel that the PSI-WPI was demanding.

### Outer setting

#### The PSI-WPI supports employees’ needs by establishing a dialogue

RCs stressed that a trusting relationship increased employees’ willingness to share information about their problems and their need of support. They also identified the involvement of the first-line manager in the RTW process as an important element of the PSI-WPI, giving the employee support outside the health care system. One RC explained “*It doesn’t matter how well we care for our patients, if we don’t have the employer with us, it is hard to get people back to work*” (RC, 8).

Employees described how important it was that the first-line manager acknowledged their situation. They wanted their first-line manager to understand that they were trying to make the best of the situation “*To get across that you’re not merely sitting around… rolling your thumbs just for the sake of it. Rather we’re actually working on it, because that’s the feeling we sometimes get when we encounter people questioning the process. You get the feeling that they don’t really understand. They haven’t really been in this situation themselves, so they don’t know what they are talking about. So that feeling… that’s something that you need to be able to communicate to them, that this is how it is…right now*” (Employee 7). In addition, employees underlined the importance of a good relationship with their RC, which increased their feelings of being supported and understood.

First-line managers appreciated the fact that the PSI-WPI enabled them to have personal contact with the RC. It gave them an opportunity to ask questions including advice on how to help their employee move forward. The first-line managers also stressed that it was important to know if an employee intended to RTW, and to have information about the employee’s current work ability. This enabled the first-line manager to help the employee not to take on too much at work; and to follow the rehabilitation plan.

The three-part meeting: RCs, employees, and first-line managers reported that the three-part meeting enabled a dialogue and a knowledge transfer to take place between them. It enabled each stakeholder’s role and responsibility to be clarified and provided an opportunity to discuss the employee’s needs during the RTW process, including work accommodations. The three-part meeting was supported by the RC communicating the purpose beforehand and providing a clear structure during the meeting. Thus, it provided an opportunity for a joint discussion and helped prevent discrepancies. During the meeting, RCs, employees, and first-line managers agreed that the RC should represent the employee but stay neutral in the discussion. RCs experienced the three-part meeting as an opportunity to gain more knowledge about the employees work situation. The employees described the importance of feeling secure and confident in the meeting which otherwise was experienced as difficult and draining. First-line managers described the three-part meeting as giving them the opportunity to show that they supported the employee in their current situation. Yet, the first-line managers reported that the meeting could be difficult for both themselves and the employee, especially if there was a conflict about how to move forward with the RTW process or if there were differing views about the cause of SA. One first-line manager felt he was poorly prepared for what the three-part meeting would involve and wished that the RC had prepared him and his employee better by explaining the aim of the meeting, how it would proceed and what would happen afterwards “*I expected a better structure, and better information and … We, it, it could have prevented it becoming quite a tuff conversation. If we had, if, if we could have steered the conversation better, or prepared both … maybe the employee and me about the purpose, what it's going to be like, what’s going to happen before and after*” (First-line manager, 5).

### Inner setting

#### The structured work method and a shared vision of how to work with SA at the PHC helps to establish the role of the RC

RCs reported that prior to the intervention, they lacked a structure for how to work “*Everybody has their own little method, how to do it. And that little method… I think one clear method is better where everyone… Everyone follows the same method, and that also make the health care more… No matter who it is it should be the same*” (RC 2). This motivated the RCs to adhere to the structure of the PSI-WPI. The RCs participation in the intervention was agreed upon with their managers enabling the RCs to set aside time for the employees in the study. Despite this, some of them reported a lack of time and available resources. The RCs also described a lack of designated coordinator resources at the PHC. Most were employed parttime in their RC role (ranging from 20 to 50%). One RC had a full-time appointment but split her working time between three PHC units. RCs often worked in a different role up to full-time – for example a district nurse or occupational therapist. They were not always able to complete their RC tasks during the allocated RC time and sometimes had to take calls and schedule meetings while working in their other role. Further, RCs also described meeting employees in both roles which could make it difficult to distinguish between meetings to plan the RTW process and meetings related to medical issues. Another problem was that some RCs worked at different workplaces on different days, which made it difficult always to have the PSI-WPI material at hand.

### Characteristics of the individual

#### The PSI-WPI created a bridge between the PHC and the workplace, conditioned by good relationships

RCs described that the PSI-WPI had become, or was starting to become, part of their daily work. The RCs felt confident in providing the intervention and followed the manual they received, except for small modifications. The role of the RC developed during the intervention, and they saw the PSI-WPI as supporting them for example in how to plan the content of and performing sessions with the employees. One RC said that she had been stagnating in her role before enrolling in the study. Participating in the PSI-WPI had given her tools that enabled her to work more actively with both employees and first-line managers “*Before we enrolled in the study, January 2018 it was, I felt very much that I was standing still in my role. So, I think this gave me tools to dare to work a little bit more actively with patients and managers*” (RC, 2). Furthermore, the RC role within the PSI-WPI was described as independent which enabled them to plan their work and schedule their time as they thought best. However, this also meant that the RC role could be a lonely one and involved a high degree of responsibility.

From the employees’ perspective, the PSI-WPI supported them in their RTW process. The PSI-WPI was described as especially valuable for those employees who needed support at the meeting with their first-line manager. Most employees experienced their contact with the RC as positive and encouraging and perceived the RC as someone who could motivate and push them to move forward with their rehabilitation. Nevertheless, two employees felt they had to little communication with their RCs which led to them to experience the relationship as non-supportive.

First-line managers described the intervention as giving them the chance to be involved in their employees’ RTW process, providing opportunities to share the manager’s view on problems related to RTW, and their ability to support the employee with, for example, workplace accommodations. The PSI-WPI was experienced by the first-line managers as creating a bridge between the workplace and health care. Some first-line managers reported a lack of experience in how to deal with a sick-listed employee and insufficient knowledge about their obligations regarding rehabilitation. In these cases, the PSI-WPI was experienced as supportive, and the first-line managers felt reassured in knowing that the RC helped to keep the rehabilitation process going by supporting their employee “*Helped to keep the process going in a way. And that it doesn’t become … that it doesn’t become the work here and the health care there but rather it’s felt like there was someone who was a bridge*” (First-line manager, 4).

## Discussion

This qualitative study had a twofold aim; the first aim was to explore the experiences of participating in a problem-solving intervention with workplace involvement aimed at reducing sickness absence in employees with common mental disorders, delivered in Swedish primary health care. The second aim was to identify facilitators of and barriers to participate in the intervention. Both aims targeted rehabilitation coordinators, employees on sickness absence, and first-line managers. Where the first aim is concerned, our findings show that all stakeholder groups reported that the PSI-WPI’s structured method supported a dialogue between them. This enabled a knowledge transfer and helped the RCs, employees and first-line managers to create a joint plan. The employees experienced the PSI-WPI as positive, helping them to take the lead and become involved in identifying problems and solutions in relation to RTW. However, some employees felt that the PSI-WPI increased their responsibility for identifying problems and solutions, which they saw as demanding. Where the second aim is concerned, participation in the PSI-WPI was facilitated by good relationships between the stakeholders and the structured process supported the employee’s active participation. First-line managers reported that the PSI-WPI provided them with early involvement in their employees’ RTW process and support from the RC regarding RTW processes. Barriers to the PSI-WPI were the time-consuming aspect reported by all three stakeholders and disagreements and conflicts between the stakeholders about for example, the cause of SA or how to move forward during the RTW process.

The structure of the PSI-WPI helped employees to take the lead in identifying problems and solutions. Previous research has shown that providing a structured approach during the RTW process can increase employees’ understanding of what is expected of them [31–33]. Understanding expectations helps employees gain control of the rehabilitation process and increases their motivation to adhere to the planned solutions [32–34]. Employees in our study also felt that the PSI-WPI provided them with a better understanding of their problems and strengthened them by having to think of possible solutions. The added value of the PSI-WPI is the strengthened ability to move from identification of problems, to trying out solutions and on to the evaluation of the process by prompting the employee’s active participation.

The PSI-WPI offered the first-line managers an opportunity to receive support from an experienced RC, for example during the three-part meeting. This gave the managers an opportunity to ask questions and learn what was expected of them. The early involvement of the first-line manager in the RTW process has been reported as an important factor for a successful RTW [8, 34, 35]. Our findings also indicate that the three-part meeting included in the PSI-WPI was experienced as supporting the collaboration between the three stakeholders. The structure of the PSI-WPI also facilitated a dialogue and a common understanding of the cause of SA and how to move forward with the RTW process.

In line with previous qualitative studies [31, 35], our study indicates that good cooperation between stakeholders, especially with emphasis on the employee-manager relationship, can facilitate the RTW process. Our results highlight the importance of allocating and dedicating time to building good relationships and cooperation between the stakeholders. Additionally, our findings also show how it is of importance to identify disagreements between the employee and first-line manager. In the first step of the PSI-WPI (“identifying problems in relation to RTW”) it is possible to identify and address disagreements and conflicting views. In such cases, the second step (“brainstorming about solutions”) and the forthcoming process can include solutions for coping with the disagreements.

All stakeholder groups reported that employees’ symptom severity, symptom fluctuation and fear of intensification of symptoms had an impact on their ability to participate in the PSI-WPI. These findings are in line with previous research focusing on RTW interventions [7, 33, 36, 37]. The impact of CMDs and related symptoms as a barrier to participation further highlights the complexity of providing interventions for employees on SA due to CMDs. In fact, most employees experience fluctuating symptoms even when starting to return to their workplaces [37]. Previous research also suggests that a reduction in CMD-related symptoms does not necessarily result in increased work ability or less SA [8]. Instead, symptoms of CMDs and RTW planning should be simultaneously addressed during the PSI-WPI, providing the employee and first-line manager with an increased understanding of problems and how to deal with them at the workplace [6, 8].

The five-step process used in the PSI-WPI has been developed and evaluated in previous studies [11, 38]. The PSI-WPI’s intention is to provide the employee with problem-solving skills. Increasing problem-solving skills may strengthen the employee’s self-efficacy, which in turn may support their RTW intention [39]. Although the PSI-WPI brought stakeholders in the RTW process together, all stakeholders reported the time-consuming aspect to be a barrier. Even if the amount of time needed to participate in the PSI-WPI was taken into consideration when planning the intervention, the impact of this aspect needs to be weighed against the positive results also reported here. The PSI-WPI potential effectiveness in reducing SA and its costs in a Swedish PHC setting will be evaluated in future research [13].

### Strengths and limitations

Our qualitative study has several strengths. A major one is the inclusion of three stakeholders (RC, employee and first-line manager) which provided us with a broad understanding of their experiences of participating in the PSI-WPI. The data allowed us to obtain explanations and descriptions of the stakeholders’ experiences of the PSI-WPI. To avoid social response bias, all participants were informed that the interviews would be conducted by independent researchers who had not been involved in the intervention. The intention was to conduct in-person interviews. However, due to the Covid-19 pandemic, the interviews with employees and first-line managers were conducted by telephone. Nevertheless, telephone interviews have been reported to have advantages due to their flexibility, the way they balance power relations, and the positive effects off not being face to face [40]. Credibility was strengthened by collecting appropriate meaning units, categorizing the codes with the help of a theoretical framework, reporting relevant quotations, and by seeking agreement among co-researchers [20]. The findings can be transferred to similar contexts because the setting is well-described, the included employees represent a variety of PHC units, and the analysis process is clearly described.

The study also has some limitations. We aimed to use a strategic sampling procedure of employees by a variation in gender, age, and work sector. However, despite several attempts to contact employees from PROSAs intervention group, only thirteen out of 72 employees agreed to participate (Fig. 2). Thus, it was not possible to achieve the strategic sampling as initially planned. One possible reason for the low response rate is that the employees still had symptoms or had RTW when contacted by the research team. The challenges of recruiting participants to research studies are well-known, adding the pressure of being on SA and struggling with a diagnosis of a CMD may well have affected their willingness to be interviewed [41, 42]. A similar study recruiting participants from a randomized controlled trial also reported a low response rate due to participants not responding or declining the invitation [43]. It may also be the case that those employees who agreed to participate in the interview had more positive experiences of PSI-WPI. However, no differences were found between the interviewed employees and the rest of the PROSA sample with regards to symptoms at baseline. The sample of RCs and employees consisted of females (no male employees consented to participating in an interview). A gender balance may have contributed to a greater variation in the data. In addition, the mean age of the RCs was 57 years which meant most had long working experience as health care professionals. There may be a difference in approaches between older and younger RCs, but that is beyond the aim of this study to elaborate on. In the PROSA trial, the employees where pre-randomized following the randomization of their RC and not informed about their allocation. Our intention was to keep the employees blinded as to their allocation during the 12-months follow-up. The interviews were therefore held after this point, which introduces the possibility of recall bias. Finally, the transferability of our results may be restricted to PHC settings.

## Conclusions

The PSI-WPI provided a structure for the stakeholders in arranging the RTW process. Seeing the workplace as an integral part of PSI-WPI by always conducting a three-part meeting enabled a dialogue that can be used to identify and address disagreements between the employee and first-line manager, to explain CMD symptoms, and how these can be handled at the workplace. We suggest allocating time towards developing good relationships as this may have positive effects on employee engagement in, and managers’ understanding of the RTW process. In addition, RCs may benefit from training in handling disagreements, although more severe conflicts probably demand other measures. Also, additional knowledge about factors in the employee’s psychosocial work environment that impairs or promotes health may increase the RCs ability to support the employee and manager. Finally, the intervention was experienced as time-consuming, a factor which needs to be taken into consideration in a large-scale implementation.

## Supplementary Information

**Additional file 1:** Interview guides.

## Data Availability

The datasets generated and analysed during the current study are not publicly available due to the Swedish ethical review regulation. Data are available upon reasonable request. Inquiries for data access should be sent to Karolinska Institutet, Institute of Environmental Medicine, Unit of Intervention and Implementation Research for Worker Health, Box 210, 171 77 Stockholm or contact the principal investigator Elisabeth Björk Brämberg, elisabeth.bjork.bramberg@ki.se, who will then contact the Swedish Ethical Review Authority for permission to openly share the data.

## Abbreviations

CFIR: Consolidated framework for implementation research
CMD: Common mental disorder
PHC: Primary health care
PSI-WPI: Problem-solving intervention with workplace involvement
RC: Rehabilitation coordinator
RTW: Return-to-work
SA: Sickness absence
