# Correction to: Experiences of participating in a problem‑solving intervention with workplace involvement in Swedish primary health care: a qualitative study from rehabilitation coordinator’s, employee’s, and manager’s perspectives

**DOI:** 10.1186/s12889-023-15990-4

**Published:** 2023-06-13

**Authors:** Ida Karlsson, Lydia Kwak, Iben Axén, Gunnar Bergström, Ute Bültmann, Kristina Holmgren, Elisabeth Björk Brämberg

**Affiliations:** 1 Institute of Environmental Medicine, Unit of Intervention and Implementation Research for Worker Health, Karolinska Institutet, Stockholm, Sweden; 2 Department of Occupational Health Sciences and Psychology, Faculty of Health and Occupational Studies, University of Gävle, Gävle, Sweden; 3 Department of Health Sciences, Community and Occupational Medicine, University of Groningen, University Medical Center Groningen, Groningen, The Netherlands; 4 Department of Health and Rehabilitation, Institute of Neuroscience and Physiology, Sahlgrenska Academy, University of Gothenburg, Gothenburg, Sweden

**Correction to**: ***BMC Public Health*** **23, 940 (2023)**

10.1186/s12889-023-15899-y

Following publication of the original article, the authors identified that the given names and family names of all authors were swapped.

The author group has been updated above and the original article has been corrected.

