# Supplementary material for: Experiences of participating in a problem-solving intervention with workplace involvement in Swedish primary health care: a qualitative study from rehabilitation coordinator's, employee's, and manager's perspectives
Source: BMC Public Health. 2023 May 24;23:940. doi: 10.1186/s12889-023-15899-y (PMC10206539; doi:10.1186/s12889-023-15899-y)
Supplement: Supplementary file 1 — Additional file 1: Interview guides. [file 12889_2023_15899_MOESM1_ESM.docx]

# Additional file 1. Interview guides

PROSA intervention consists of 1) a meeting between the employee and the RC at the primary care centre to map his/her situation and problems and solutions for return to work, 2) a three-part meeting between the RC, employee and first-line manager and 3) a follow-up, either by phone or in person between the RC and employee (and potentially with the first-line manager).

**Interview guide for RCs**

**Intervention characteristics**

**Intervention source**

-Could you describe how the decision to participate in the PROSA-study was made?

-Who took the decision?

**Relative advantage**

-Would you say the intervention has become a part of your daily work at the primary care centre?

-If yes, please explain how!

-Could you describe factors you think facilitated giving the intervention?

-In what way have they facilitated?

**Adaptability**

-Could you tell me about how you have been working with giving the PROSA-intervention?

-Could you tell me about why you have shaped your work with the intervention in this way?

-Have you needed to make any changes in the intervention to make it work?

-If so, which changes?

-Have you had to make any changes in your work to be able to give the intervention?

-If you could have changed something in the PROSA-intervention, what would that have been?

**Complexity**

-Could you describe factors you think have hindered giving the intervention?

-In what way have they hindered?

**Design packaging and quality**

-What do you think about the quality of the worksheets that were included in the intervention?

**Outer setting**

**Patient needs and resources**

-How do you perceive the intervention?

-Do you think the intervention has been supportive for the patients included in the study?

-What feedback have you received from your patients (participants)?

-What do you think has hindered the participants from participating in the PROSA-study?

-How do you perceive the first-line manager’s contribution?

-Could you describe if you think the intervention has been supportive for the first-line manager?

-What feedback have you received from the first-line managers/managers that you have met?

**Inner setting**

**Structural characteristics**

-Could you tell me about what support has been valuable for you to be able to give the intervention? Eg, support from your employer, colleagues at the primary care centre, network for RCs etc.

-Do you feel that the set-up at the primary care centre has had an impact on the ability to give the intervention?

-In what way has it facilitated/hindered?

-How have you worked with these challenges?

**Networks and communication**

-Could you describe your network (RCs, process leaders etc)?

-What support have you had from your network in for example carrying out three-part meetings?

**Compatibility**

-Could you tell me whether (and in which case, how) the PROSA-intervention deviates from how you usually work as a rehabilitation coordinator?

-Could you describe how well you think the intervention is designed to meet the needs of your patients who are on sick leave for mental illnesses?

-Could you describe how well you think the intervention coincides with the norms and values at your primary care centre?

**Available resources**

-What resources did you have to give the PROSA-intervention?

**Access to knowledge and information**

-If you think back on the training for PROSA, what is your impression?

-Wat was positive? Do you feel anything was missed?

**Characteristics of individuals**

**Knowledge and beliefs about the intervention**

-What is your attitude/position towards the PROSA-intervention?

-What do you think the aim of the intervention was?

**Self-efficacy**

-Do you believe that you have provided the intervention in an appropriate manner?

**Process**

**Executing**

-Have you used any other material?

**Interview guide for Employees**

The PROSA-intervention consists of 1) the employee meeting the RC at the primary care centre to map his/her situation and possibilities of return to work, 2) a three-part meeting with your first-line manager, you as employee and the RC, and 3) a follow-up meeting together with the RC, whit your first-line manager possibly participating, either physically or by phone.

**Intervention characteristics**

**Intervention source**

-Could you tell me about your thoughts when you decided to participate in the PROSA-study?

**Relative advantage**

-Could you describe factors you think have facilitated taking part in the PROSA-intervention

-In what way have they facilitated?

**Complexity**

-Could you describe what factors you think have hindered your participation in the PROSA-intervention?

-In what way have they hindered?

**Adaptability**

-If you could have changed something in the PROSA-intervention, what would that be?

**Design packaging and quality**

-Could you tell me how well the intervention was designed to meet your needs when you were on sickness absence for a mental disorder?

**Outer setting**

**Patient needs and resources**

-How do you think your first-line manager perceived the intervention?

-Can you tell me if you believe the intervention has been supportive for your first-line manager?

**Inner setting**

**Structural characteristics**

-How do you see the meeting(s) with the RC at the primary care centre?

-What do you think about the meeting(s) with the RC, you and your first-line manager?

-What was the result of the meeting with the RC and your first-line manager?

-How did the evaluation meeting with the RC take place?

**Characteristics of individuals**

**Knowledge and beliefs about the intervention**

-How do you see your participation in the study?

-Could you tell me about what the PROSA-intervention has meant to you?

**Process**

**Executing**

-What parts of the intervention (according to the above definition of the intervention) did you participate in?

**Interview guide for First-line managers**

The PROSA-intervention consists of 1) the employee meeting the RC at the primary care centre to map his/her situation and possibilities of return to work, 2) a three-part meeting with your employee, you as first-line manager and the RC, and 3) a follow-up, in which you as first-line manager possibly have participated, either physically or by phone.

**Intervention characteristics**

**Relative advantage**

-Can you describe which factors you think have facilitated your participation in the three-part meeting?

-In what way have they facilitated?

**Adaptability**

-If you could have changed something in the three-part meeting or the PROSA-intervention, what would that be?

**Complexity**

-Can you describe which factors have hindered or facilitated you to participate in the three-part meeting?

-In what way have they hindered or facilitated?

**Design packaging and quality**

-Can you tell us how it was to participate in the three-part meeting with your employee and the RC?

-What worked well? What was difficult?

**Outer setting**

**Patient needs and resources**

-How do you think that your employee perceived the three-part meeting?

**Inner setting**

**Available resources**

-Can you tell us what support has been valuable in connection with your employee’s sickness absence? For example, support from your manager, HR, your colleagues, the RC, or other staff from the primary care centre?

-In what way was that support important for you?

**Characteristics of individuals**

**Knowledge and beliefs about the intervention**

-Can you describe what your thoughts were when you were asked to participate in a three-part meeting with your employee and the RC at the primary care centre?

-Can you describe how well you think the three-part meeting was designed to meet your needs as a first-line manager/manager?

-Can you describe whether you think that the intervention provides support to the first-line manager?

**Process**

**Executing**

-What parts of the intervention (according to the definition above) did you take part in?
